# Dietary Diversity in Children with Autism Spectrum Disorders, Feeding Difficulties, and Sensory Issues Related to Eating—A Case–Control Study

**DOI:** 10.3390/nu18183061

**Published:** 2026-09-19

**Authors:** Martina Grot-Nigowska, Oskar Kowalski, Agnieszka Białek-Dratwa

**Affiliations:** 1 Doctoral School, Medical University of Silesia in Katowice, 15 Poniatowskiego St., 40-055 Katowice, Poland; 2 Department of Human Nutrition, Division of Dietetics, School of Public Health in Bytom, Medical University of Silesia in Katowice, Building IVA, SUM Campus, 19 Dr. Henryk Jordan St., Zabrze-Rokitnica, 41-808 Zabrze, Poland

**Keywords:** dietary diversity score, dietary selectivity, ASD, children

## Abstract

**Background/Objectives:** Pediatric feeding disorders are common in children with autism spectrum disorder (ASD) and may result from sensory processing differences, contributing to reduced dietary diversity and increasing the risk of nutritional deficiencies or excessive body weight. This study assessed whether children with ASD exhibit lower dietary diversity than typically developing children. **Methods:** A clinical case–control study included 181 children aged 2–10 years (ASD: *n* = 85; controls: *n* = 96). Children with ASD had a confirmed diagnosis, while controls had no neurodevelopmental disorders. Dietary diversity was assessed using the Dietary Diversity Score (DDS), based on a 24 h dietary recall completed by parents or legal guardians. Foods were classified into nine food groups. DDS was categorized as low (≤4 groups), moderate (5–6), or high (≥7). **Results:** Mean total DDS was comparable between groups (ASD: 6; controls: 5). Children with ASD consumed legumes and oilseeds more frequently, whereas controls consumed dark green leafy vegetables more often. Low DDS was more prevalent in the ASD group (32.9% vs. 15.6%), while moderate DDS predominated in controls (64.6% vs. 32.9%). High DDS was also more common in children with ASD (34.1% vs. 19.8%), indicating substantial heterogeneity in dietary patterns. Dietary diversity was significantly associated with sensory characteristics, particularly acceptance of food texture and temperature. **Conclusions:** The study results indicate a heterogeneous nature of dietary diversity among children with ASD, without confirming a generally reduced level. After adjusting for age, sex, and BMI, ASD group membership was associated with DDS scores. Individual and sensory-related dietary factors should be taken into account when addressing nutritional needs.

## 1. Introduction

In children with autism spectrum disorder (ASD), feeding difficulties and pediatric feeding disorders (PFDs) occur significantly more frequently than in neurotypical children. Depending on the diagnostic criteria used and the characteristics of the study population, their prevalence is estimated at 30–84% [1]. According to the consensus definition proposed by Goday et al., PFD is defined as an age-inappropriate disturbance in oral food intake that persists for at least two weeks and is accompanied by dysfunction in at least one of four areas: medical, nutritional, feeding skills, or psychosocial. This definition emphasizes the multifactorial nature of feeding difficulties and highlights the need for comprehensive, interdisciplinary assessment and management [1].

In children with ASD, feeding difficulties may arise from the interaction of neurodevelopmental, sensory, behavioral, nutritional, gastrointestinal, and environmental factors [1]. Food selectivity, rigid eating patterns, refusal of specific foods, and sensory hypersensitivity to food characteristics such as texture, taste, smell, temperature, or appearance may substantially influence food acceptance and the range of foods consumed. These characteristics may contribute to repetitive food choices and avoidance of particular food groups, potentially resulting in reduced dietary variety and an increased risk of nutritional inadequacy [2,3]. Thus, feeding difficulties should not be considered solely as behavioral phenomena; they may also have nutritional consequences that are relevant to the assessment and management of children with ASD.

Sensory processing may represent one of the mechanisms linking feeding difficulties with reduced dietary variety. Atypical sensory responses are frequently reported in children with ASD and may influence the acceptance or rejection of foods according to their sensory properties [4,5]. Consequently, children who experience food-related sensory stimuli as aversive may restrict their food repertoire, whereas other children with ASD may display fewer feeding difficulties despite having the same neurodevelopmental diagnosis. This suggests that dietary diversity may vary considerably within the ASD population and that the presence of ASD alone may not sufficiently explain individual differences in eating patterns.

The eating habits of children with ASD have frequently been described as less diverse than those of neurotypical children, with selective eating and sensory-related food avoidance often associated with lower consumption of vegetables, fruits, and selected protein-rich foods [3,5]. Differences in nutrient intake have also been reported, including lower intakes of selected vitamins and calcium, although findings are not consistent across studies and differences in magnesium, iron, zinc, phosphorus, and sodium intake have not been consistently demonstrated [3,5]. These inconsistencies may reflect differences in age, dietary assessment methods, sample characteristics, diagnostic criteria, and the degree of feeding selectivity across study populations. Therefore, although reduced dietary variety is frequently reported in ASD, the magnitude and determinants of this phenomenon remain insufficiently characterized.

Dietary diversity can be assessed using the Dietary Diversity Score (DDS), which is based on the number of predefined food groups consumed during a specified period [6,7,8]. The DDS provides an overall measure of the variety of foods consumed and may be useful for identifying restricted dietary patterns. However, dietary diversity should not be equated with overall diet quality or nutritional adequacy, as consumption of a larger number of food groups does not necessarily indicate appropriate quantities, nutrient density, or adherence to dietary recommendations. In children with ASD, the DDS may nevertheless provide a useful complementary measure for examining whether feeding difficulties and sensory characteristics are accompanied by a narrower range of consumed food groups [6,7,8].

Importantly, previous studies have not consistently demonstrated that all children with ASD exhibit the same degree of dietary restriction. While some studies report markedly limited food repertoires and lower consumption of selected food groups, other children with ASD may consume a relatively diverse diet [3,5,6,7,8]. This heterogeneity suggests that feeding difficulties, sensory characteristics, and dietary diversity may represent related but distinct dimensions of eating behavior. Consequently, examining only the presence or absence of feeding difficulties may not adequately capture differences in dietary patterns within the ASD population. A more comprehensive approach that considers dietary diversity together with feeding difficulties and selected sensory characteristics may provide a better understanding of these individual differences.

In the most severe cases, persistent food selectivity and substantial restriction of dietary variety may be associated with clinically significant nutritional or psychosocial consequences and require differential consideration of Avoidant/Restrictive Food Intake Disorder (ARFID). Food selectivity, however, is not synonymous with ARFID. According to the Diagnostic and Statistical Manual of Mental Disorders (DSM−5), ARFID is diagnosed when restricted food intake is associated with clinically significant consequences, including nutritional deficiencies, weight loss or failure to achieve expected growth, dependence on oral nutritional supplements or enteral feeding, or significant psychosocial impairment [9,10,11]. ARFID has been reported more frequently among children with ASD than in the general population, further emphasizing the importance of distinguishing common selective eating from clinically significant restrictive eating disorders [9,10,11].

Despite growing evidence concerning feeding difficulties, sensory processing, and nutritional characteristics in ASD, relatively few studies have examined these domains simultaneously in relation to dietary diversity. In particular, it remains unclear whether differences in dietary diversity between children with and without ASD are accompanied by differences in feeding difficulties and selected sensory characteristics, and whether the relationship between feeding difficulties and dietary diversity persists when demographic and anthropometric characteristics are considered. Addressing these questions may help clarify whether dietary diversity represents a relatively uniform characteristic of children with ASD or reflects heterogeneous dietary profiles associated with individual feeding characteristics.

Therefore, the aim of this study was to assess dietary diversity, measured using the Dietary Diversity Score (DDS), in children with ASD compared with children without diagnosed neurodevelopmental disorders, and to examine dietary diversity in relation to feeding difficulties and selected sensory characteristics associated with food intake. Based on the previous literature, we hypothesized that (1) children with ASD would have greater feeding difficulties than children without diagnosed neurodevelopmental disorders; (2) children with ASD would be more likely to exhibit lower dietary diversity; (3) greater feeding difficulties and more pronounced sensory-related food characteristics would be associated with lower dietary diversity; At the same time, given the heterogeneity reported in the literature, we did not assume that all children with ASD would exhibit low dietary diversity.

## 2. Materials and Methods

### 2.1. Study Protocol

The study was conducted between November 2023 and April 2024 at two medical facilities. Participants were recruited at two facilities with different areas of specialization. One of them provided nutritional counseling to children without diagnosed neurodevelopmental disorders, while the other provided diagnosis, therapy, and rehabilitation for children with neurodevelopmental disorders. Children from both groups were referred for consultation at the clinic by the facilities’ medical staff (a physician, clinical dietitian, or pediatrician) in accordance with the centers’ routine work procedures. Before participating in the study, parents or legal guardians were informed about its organization, procedure, the rules governing the processing of personal data, and the voluntary nature of participation. The information provided to participants was general in nature and did not include specific study objectives or hypotheses, in order to minimize the risk of influencing their responses. Subsequently, informed, written consent was obtained from parents or legal guardians for the child’s participation in the study. Sample selection complied with the ethical principles of research involving human subjects, in accordance with the Declaration of Helsinki. Approval was obtained from the Bioethics Committee of the Medical University of Silesia in Katowice to conduct a case–control study. The Bioethics Committee approved a study titled “Feeding Disorders and Oral Hypersensitivity in the Context of Sensory-Based Feeding in Patients with Neurodevelopmental Disorders” (Resolution No. PCN/CBN/0052/KB1/142/22/23; dated 28 February 2023).

### 2.2. Group Selection

A total of 181 children aged 2 to 10 years were enrolled in the study and divided into a control group (*n* = 96) and a study group (*n* = 85). In the control group, girls accounted for 58.33% (*n* = 56) and boys for 41.67% (*n* = 40). In contrast, in the experimental group, girls accounted for 32.94% (*n* = 28), and boys for 67.06% (*n* = 57).

The diagnosis of autism spectrum disorder (ASD) was made by a child and adolescent psychiatrist in accordance with the diagnostic criteria outlined in the fifth edition of the Diagnostic and Statistical Manual of Mental Disorders (DSM−5). The diagnosis was confirmed by medical records provided by the child’s parents or guardians and served as the criterion for inclusion in the study. Children were included in the study group based on medical records provided by their parents or legal guardians confirming the diagnosis of ASD. These medical records served as the basis for verifying that the inclusion criteria for the study were met.

The control group consisted of children from the general population of the Silesian Voivodeship, recruited from randomly selected educational institutions, including daycare centers, preschools, and elementary schools. The children in the control group were examined at a dietary counseling clinic, where a standard assessment of their nutritional status and eating habits was conducted. It should be emphasized that the children assigned to the control group were not patients of the clinic where the study was conducted. The absence of a diagnosis of ASD or other neurodevelopmental disorders was confirmed based on interviews with parents or legal guardians and information contained in available medical records from physicians and/or other healthcare professionals working at the respective facility. For children enrolled in the control group, parents did not report a diagnosis of autism spectrum disorder or other neurodevelopmental disorders, and the children had no medical records confirming such a diagnosis. Both groups included children aged 2 to 10 years whose parents or legal guardians provided written informed consent for participation in the study. Children with incomplete documentation that prevented unambiguous classification into the appropriate group, as well as children who did not meet the established inclusion criteria, were excluded from the study.

Participants were selected using convenience sampling, which may have led to selection bias. This method is a type of non-random sampling based on the availability of participants who meet the study’s inclusion criteria. Consequently, the study sample may not be fully representative of the entire population of children on the autism spectrum, which limits the generalizability of the results. Participants were recruited using convenience sampling from two clinical centers with different profiles. The use of convenience sampling may have carried a risk of selection bias, as participants were not randomly selected from the target population. In particular, children recruited from clinical centers may differ from children in the general population in terms of clinical characteristics, eating behaviors, and access to specialized care. For this reason, the results should be interpreted primarily in relation to the study population, and any generalization to all children aged 2–10 years should be treated with caution. To mitigate the potential impact of inter-group differences on the analyzed results, a multivariable linear regression analysis was additionally performed, accounting for age, sex, and BMI as potential confounders. The limitations associated with the use of convenience sampling were also addressed in the interpretation of Section 3 and Section 7.

### 2.3. Research Tools

#### 2.3.1. Body Mass Index (BMI)

The nutritional status of children was assessed based on the Body Mass Index (BMI), calculated as the quotient of body weight in kilograms divided by the square of height in meters (kg/m^2^). Due to changes in BMI values that occur with age and differences based on sex, the obtained BMI values were compared to the centile charts developed as part of the OLA and OLAF projects, which serve as Polish reference standards for the population of children and adolescents. The centile charts allow for the assessment of a child’s position relative to a population of peers of the same sex and age [12].

Based on the centile position obtained, nutritional status was classified according to current criteria. A BMI below the 5th percentile was considered underweight; a BMI between the 5th and just below the 85th percentile was considered normal weight; a BMI between the 85th and just below the 97th percentile was considered overweight; and obesity was diagnosed for a BMI equal to or exceeding the 97th percentile [12].

#### 2.3.2. Dietary Diversity Score (DDS)

The DDS index, based on a 24 h dietary recall of the child’s food intake, was used to assess dietary diversity. The DDS was calculated in accordance with the methodology recommended by the Food and Agriculture Organization of the United Nations (FAO). The DDS reflected the variety of food groups consumed on the assessed day and was not intended to represent habitual dietary diversity over an extended period. Based on a dietary interview conducted with a parent or legal guardian, a 24 h record of the child’s food intake was obtained, covering nine food groups: (1) grains, (2) dark green leafy vegetables, (3) root and tuber vegetables, (4) other vegetables, (5) fruits and fruit juices, (6) legumes and oilseeds, (7) meat, fish and poultry, (8) milk and dairy products, and (9) eggs. The results for each participant were individually recorded by a dietitian from the research team [13]. One point was assigned when at least half a serving of one food item from a given food group was consumed. The DDS was calculated as the total number of food groups consumed, resulting in a possible score ranging from 0 to 9. For the purposes of the present study, DDS values were categorized into three levels: low (≤4 food groups), moderate (5–6 food groups), and high (≥7 food groups), based on previously published classifications [13,14]. These cut-off values were used as analytical categories and should not be interpreted as universally established FAO thresholds or as clinically validated cut-offs specific to children with ASD.

#### 2.3.3. Montreal Children’s Hospital Food Scale (MCH-FS)

To identify behavioral and sensory difficulties, the Polish version of the Montreal Children’s Hospital Feeding Scale (MCH-FS) was used. In this study, we used a questionnaire developed as a result of a previous process of translation, cultural adaptation, and validation conducted by other authors. The questionnaire is intended for children aged 6 months to 6 years [4,15]. Given the specific characteristics of the study group and the nature of the feeding difficulties being assessed, the MCH-FS was also applied to children aged 2 to 10 years. The questionnaire consists of 14 questions concerning, among other things, texture acceptance, meal duration, feeding style, and parental stress levels. It includes the following questions: How would you rate your child’s eating pattern? How concerned are you about your child’s eating pattern? How would you rate your child’s appetite (sense of hunger)? At what point during a meal does your child start refusing to eat? How long do your child’s meals last (in minutes)? How would you rate your child’s behavior during meals? Does your child choke, gag, spit up, or vomit after eating certain foods? Does your child hold food in their mouth without swallowing? Do you have to follow your child around or distract them (with toys, TV) to get them to eat? Do you have to force your child to eat or drink? How would you rate your child’s chewing (or sucking) skills? How do you assess your child’s growth (weight, height)? How does feeding your child affect your relationship with them? How does feeding your child affect your family relationships? The MCH-FS questionnaire assesses the following feeding characteristics: oral motor skills, oral sensitivity, and appetite, as well as mothers’ anxiety during feeding, behaviors during meals, the mother’s coping strategies, and the family’s reactions to the feeding process. Each question is rated on a 7-point Likert scale [4,14]. Interpretation of the MCH-FS indicates that scores ranging from 14 to 45 points indicate no feeding difficulties, scores of 46–52 points indicate mild feeding difficulties, scores of 53–58 points indicate moderate feeding difficulties, and scores of 59 points or higher indicate severe feeding difficulties. The Polish version of the Montreal Children’s Hospital Feeding Scale (MCH-FS), validated for children aged 6 months to 6 years, was used to assess feeding difficulties. The instrument was applied consistently across the study sample, including children older than 6 years, to allow the same measure to be used for comparison between the ASD and control groups. However, its use in children aged 7–10 years represents an extension beyond the validated age range, and the results obtained in this age group should therefore be interpreted with caution. To assess the robustness of the findings, an additional sensitivity analysis was performed in children aged ≤6 years.

### 2.4. Statistical Analysis

Statistical analysis was performed using Statistica v. 13.3 (StatSoft Inc., Tulsa, OK, USA). The normality of the variable distributions was assessed using the Shapiro–Wilk test. The analyzed variables did not follow a normal distribution (*p* < 0.05); therefore, nonparametric statistical methods were used. The use of nonparametric tests allowed for more reliable results when the assumptions of parametric tests were not met. Numerical data are presented as the median and interquartile range, where Q1 denotes the lower quartile (25th percentile) and Q3 the upper quartile (75th percentile), as well as the minimum (Min) and maximum (Max) values. The Mann–Whitney U test was used to compare two independent groups (the study group and the control group) with respect to measurable variables. Student’s *t*-test for independent samples was used to compare the means of quantitative variables between the two independent groups. Feeding difficulty and the severity of food selectivity were assessed using the MCH-FS. The total score on the scale was treated as a continuous variable. To determine the practical significance of the differences between the study group and the control group, the effect size was calculated using Cohen’s d. The d value was calculated as the quotient of the difference between the arithmetic means of the compared groups and the total standard deviation. The effect size was interpreted according to the criteria proposed by Jacob Cohen: d = 0.20—small effect, d = 0.50—medium effect, d = 0.80—large effect. Cohen’s d was calculated for the total score on the MCH-FS to assess the magnitude of the difference between the study group and the control group, regardless of the statistical significance of the test. The relationships between the MCH-FS score and demographic variables as well as the dietary diversity score (DDS) were analyzed using Spearman’s rank correlation. Dietary diversity was assessed using the DDS, calculated as the number of food groups consumed within a 24 h period (range 0–9). The DDS was analyzed as a continuous variable. Differences in DDS values between the study group and the control group were assessed using the Mann–Whitney U test. For the purposes of analysis, categorical variables (MCH-FS interpretation and DDS) were converted to binary form by comparing the selected category with the remaining levels. Analyses were conducted using 2 × 2 contingency tables. The strength of the association between variables was assessed using the odds ratio (OR) along with the 95% confidence interval (95% CI). Statistical significance was assessed using the NW chi-square test. For categorical variables—food product groups classified by DDS index (low, medium, high)—Fisher’s exact test was used due to the small sample sizes in the analyzed subgroups. To identify factors associated with the severity of feeding difficulties and dietary diversity, a linear regression analysis was conducted. In the linear regression analysis, regression coefficients (β) were reported along with their corresponding standard errors (SEs) and significance levels (*p*). Model fit was assessed based on the coefficient of determination (R^2^). The level of statistical significance in this study was set at *p* ≤ 0.05. To assess whether the observed differences in eating behaviors between children with autism spectrum disorder (ASD) and the control group were independent of selected potential confounders, a multivariable linear regression analysis was performed. A separate regression model was developed for each outcome analyzed. Group membership (ASD vs. control group) served as the primary independent variable, while age, sex, and BMI were included as potential confounders. In the first model, the dependent variable was the Montreal Children’s Hospital Feeding Scale (MCH-FS) score, whereas in the second model, it was the total Dietary Diversity Score (DDS). Regression coefficients are presented alongside 95% confidence intervals and *p*-values. Model fit was assessed using the coefficient of determination (R^2^) and adjusted R^2^. Prior to the analysis, linear regression assumptions—including the linearity of relationships, homoscedasticity of residuals, and multicollinearity among independent variables—were evaluated. Multicollinearity was assessed using the variance inflation factor (VIF). Due to observed heteroscedasticity, HC3 robust standard errors were employed. All statistical tests were two-tailed, and the level of statistical significance was set at *p* < 0.05.

Analyses of individual questionnaire items, food groups, and age-specific comparisons were considered secondary and exploratory. No formal adjustment for multiple comparisons was applied; therefore, these findings were interpreted cautiously, taking into account the increased risk of Type I error.

## 3. Results

### 3.1. Characteristics of the Study Sample

The study compared a group of children with ASD (*n* = 85) with a control group (*n* = 96), revealing statistically significant differences in demographic characteristics, anthropometric parameters, and nutritional status. Detailed data are presented in Table 1.

### 3.2. Analysis of Dietary Selectivity

The MCH-FS score in the study group (*n* = 85) was Me (median) = 53 points (Q1–Q3: 49–57), with a minimum score of 41 points and a maximum score of 72 points, while in the control group (*n* = 96), Me (median) = 54 points (Q1–Q3: 53–55.5), minimum value = 41 points, maximum value = 72 points. Analysis using the Mann–Whitney U test did not reveal any statistically significant differences between the groups (U = 3441.50; Z = −1.81; *p* = 0.068). In the study group, the median MCH-FS score was similar to that of the control group. Differences between the groups were not statistically significant when considering the child’s age (2 to 10 years) and the total MCH-FS score (U = 87.50; *p* = 0.397). An analysis of the relationship between the child’s age and the MCH-FS score using Spearman’s rank correlation coefficient did not reveal any significant correlations in either the study group (r_s_ = 0.039, *p* > 0.05) or the control group (r_s_ = 0.068, *p* > 0.05). In all age categories analyzed, the medians of the MCH-FS scores were similar in both groups. In the study group, the medians ranged from 52.00 to 54.00 points, while in the control group, they ranged from 53.00 to 57.50 points.

Among 2-year-olds, the median MCH-FS score was 53.50 points (Q1–Q3: 52.00–55.00) in the study group (*n* = 2) and 54.00 points (Q1–Q3: 51.50–59.00) in the control group (*n* = 4) (*p* = 1.000). In the group of 3-year-olds, there were no children in the study group, while in the control group (*n* = 6) the median was 54.50 points (Q1–Q3: 49.00–64.00); the difference was also not statistically significant (*p* = 1.000).

Among 4-year-olds, the median was 53.50 points (Q1–Q3: 42.00–55.00) in the study group (*n* = 6) and 53.00 points (Q1–Q3: 52.00–55.00) in the control group (*n* = 5) (*p* = 0.927). Among 5-year-olds, the median was 54.00 points (Q1–Q3: 47.00–54.50) in the study group (*n* = 12) and 57.50 points (Q1–Q3: 49.00–61.50) in the control group (*n* = 4) (*p* = 0.220). In the group of 6-year-olds, the medians were 52.00 points (Q1–Q3: 49.00–54.00) for the study group (*n* = 18) and (control group: *n* = 2) 53.50 points (Q1–Q3: 53.00–54.00) (*p* = 0.375).

Among 7-year-olds, the median was 54.00 points (Q1–Q3: 49.00–61.00) in the study group (*n* = 15) and 55.00 points (Q1–Q3: 53.00–57.00) in the control group (*n* = 9) (*p* = 0.905). In the group of 8-year-old children, the medians were 54.00 points (Q1–Q3: 41.00–59.00) and 53.50 points (Q1–Q3: 50.50–55.00), respectively (*p* = 0.907). Among 9-year-olds, the median was 53.00 points (Q1–Q3: 49.00–57.00) in the study group and 53.50 points (Q1–Q3: 52.00–54.00) in the control group (*p* = 0.845). In contrast, among 10-year-olds, the median MCH-FS score was identical in both groups and amounted to 54.00 points, with quartile ranges of 49.00–58.00 and 54.00–56.00, respectively (*p* = 0.709).

A comparative analysis using the Mann–Whitney U test showed that in none of the analyzed age categories were statistically significant differences found between the study group and the control group (in all cases, *p* > 0.05). The results indicate that the child’s age did not influence the differences in MCH-FS scores between the study and control groups. The descriptive analysis of the MCH-FS used the mean, standard deviation, and median for each question. Differences were observed in six responses between the study group and the control group regarding specific items on the MCH-FS. In the study group, compared to the control group, differences in responses were observed regarding the timing of meal refusals (mean 2.64 ± 2.01, median = 1 vs. mean 5.47 ± 2.27, median = 7), assessment of meal progression (mean 3.15 ± 1.84, median = 2 vs. mean 5.73 ± 1.75, median = 7), appetite level (mean 3.66 ± 2.15, median = 3 vs. mean 5.89 ± 1.71, median = 7), the need to be forced to eat and drink (mean 3.21 ± 2.39, median = 2 vs. mean 5.91 ± 1.88, median = 7), choking, gagging, and spitting out specific foods during meals (mean 4.67 ± 2.19, median = 6 vs. mean 1.93 ± 1.74, median = 1), and mealtime behaviors (mean 5.28 ± 2.03, median = 6 vs. mean 2.29 ± 1.61, median = 2). The study group had a higher mean score for feeding difficulty symptoms, along with greater variability in the scores. The mean total score on the MCH-FS was comparable in both groups (study group = 52.32 vs. control group = 54.06); however, the standard deviation was higher in the study group (SD = 7.01 vs. SD = 4.09), which may indicate greater variation in the severity of feeding difficulties among children on the autism spectrum. In contrast, the effect size of the difference in the total score on the MCH-FS between the experimental and control groups was small (d = 0.30), indicating that the difference analyzed has little practical significance. The distribution of responses to the descriptive questions for the MCH-FS, including the median, mean, and standard deviation, broken down by the experimental and control groups, is presented in Table 2.

Multivariable linear regression was used to determine the association between ASD status and MCH-FS scores, adjusting for potential confounders. The model included group membership, age, sex, and BMI. Group membership (ASD vs. control) was not significantly associated with MCH-FS scores (β = −1.91; 95% CI: −4.07–0.05; *p* = 0.056). Similarly, age (β = −0.11; *p* = 0.623), sex (β = 1.05; *p* = 0.256), and BMI (β = 0.02; *p* = 0.836) showed no significant association with MCH-FS scores. The model explained 3.4% of the variance in MCH-FS scores (R^2^ = 0.034; adjusted R^2^ = 0.012).

Due to the limited age range of the validation of the Polish version of the Montreal Children’s Hospital Feeding Scale (MCH-FS), an additional sensitivity analysis was conducted, restricted to children aged ≤ 6 years. The analysis included 59 participants, comprising 38 children with ASD and 21 children in the control group. A multivariable linear regression analysis was performed, accounting for group membership, age, sex, and BMI. In the analysis restricted to children aged ≤ 6 years, ASD group membership was not significantly associated with the MCH-FS score (β = −3.34; 95% CI: −7.50–0.82; *p* = 0.116). Age (β = −0.45; *p* = 0.517), sex (β = 1.04; *p* = 0.569), and BMI (β = −0.03; *p* = 0.900) also showed no significant association with the MCH-FS score. The model explained 7.8% of the variance in the MCH-FS score (R^2^ = 0.078; adjusted R^2^ = 0.009). The direction of the association between ASD group membership and the MCH-FS score was consistent with the result obtained in the analysis of the entire study population; however, in the analysis restricted to children aged ≤ 6 years, it did not reach statistical significance.

### 3.3. Analysis of the Dietary Diversity Index

The DDS in the study group (*n* = 85) was Me (median) = 6 points (Q1–Q3: 4–7), with a minimum value of 2 points and a maximum value of 9 points, while in the control group (*n* = 96), Me (median) = 5 points (Q1–Q3: 5–5.5), minimum value = 2 points, maximum value = 9 points. Analysis using the Mann–Whitney U test did not reveal any statistically significant differences between the groups (U = 3599.50; Z = 1.36; *p* = 0.172). After adjustment for age, sex, and BMI, ASD group membership was significantly associated with a higher DDS (β = 1.07, 95% CI 0.44–1.70, *p* < 0.001). The adjusted model explained 7.8% of the variance in the DDS (adjusted R^2^ = 0.057), indicating that the included predictors accounted for only a modest proportion of individual differences in dietary diversity.

In the study group, the median DDS was similar to that of the control group. Differences between the groups were not statistically significant in the study population of children aged 2–10 years with respect to the total DDS (U = 86.50; *p* = 0.359). An analysis of the relationship between the child’s age and the DDS index using Spearman’s rank correlation coefficient revealed a moderate positive correlation in the study group (r_s_ = 0.387, *p* ≤ 0.05), whereas a negative, statistically nonsignificant correlation was observed in the control group (r_s_ = −0.161, *p* > 0.05). This indicates that, as children with ASD aged, there was a tendency for them to achieve higher DDS scores, whereas no such trend was observed in the control group.

DDS scale results for the study and control groups across specific age categories are presented in Table 3. In the group of children with ASD, median DDS scores range from 4 to 7 points, with the highest values observed in the older age groups. Medians in the control group fell within the 5–6 point range. The highest median in the ASD group was found among 10-year-olds (7 points; Q1–Q3: 7–8), whereas the median for this age category in the control group was 5 points (Q1–Q3: 5–5). Given the basic characteristics of some age subgroups, these values should be treated as descriptive features of the score distributions. To assess the relationship between group membership and the DDS, with age included as a continuous variable, a multivariable linear regression analysis was performed. The model included group membership, age, sex, and BMI. Age did not show a significant association with the DDS (β = −0.05; *p* = 0.395). Median DDS values varied across age groups; however, the age-specific subgroup sizes were small, with some subgroups including only a few participants. Therefore, formal between-group comparisons within individual age categories were considered exploratory and were interpreted with caution. The statistically significant difference observed among 10-year-old participants should be regarded as an exploratory finding rather than evidence of a robust age-specific effect, particularly because age-stratified analyses were not prespecified as primary analyses.

The findings did not indicate a uniform reduction in dietary diversity among children with ASD. Although no statistically significant between-group difference was observed in the continuous DDS (median 6 vs. 5, *p* = 0.172), the categorical analysis showed a more heterogeneous distribution of DDS in the ASD group, with children represented more frequently in both the low- and high-DDS categories compared with the control group. These findings support the need for individualized assessment of dietary diversity and nutritional needs in children with ASD.

The descriptive analysis of the dietary diversity score used the mean, median, and standard deviation for each food group. The analysis revealed significantly higher index values for the groups comprising other vegetables (*p* < 0.001), legumes and oilseeds (*p* < 0.001), and milk and dairy products (*p* = 0.010) in the study group compared with the control group. In contrast, significantly higher values of the index for dark green leafy vegetables were observed in the control group (*p* < 0.001). For the remaining food product groups, no statistically significant differences were found between the groups (*p* > 0.05). The distribution of intake for individual food groups, along with their median, mean, and standard deviation values, broken down by the study group and the control group, is presented in Table 4.

Regarding the total DDS, membership in the ASD group remained significantly associated with the DDS after adjusting for age, sex, and BMI (β = 1.07; 95% CI: 0.44–1.70; *p* < 0.001). This indicates that, with the other variables included in the model held constant, children with ASD scored, on average, approximately 1.07 points higher on the DDS compared to children in the control group. BMI also showed a statistically significant association with the DDS (β = 0.09; 95% CI: 0.04–0.15; *p* < 0.001). Age (β = −0.05; *p* = 0.395) and sex (β = −0.53; *p* = 0.055) did not reach statistical significance. The model explained 7.8% of the variance in the DDS (R^2^ = 0.078; adjusted R^2^ = 0.057).

A low dietary diversity score was observed in 32.94% of the children in the study group (*n* = 28) and in 15.63% of the children in the control group (*n* = 15). An average dietary diversity score was observed in 32.94% (*n* = 28) of the children in the study group and 64.58% (*n* = 62) of the children in the control group, respectively. A high level of dietary quality was observed in 34.12% (*n* = 29) of children with autism spectrum disorders and in 19.79% (*n* = 19) of children in the control group. Consumption of grain products (white wheat or rye bread, wheat-rye bread, toast, plain rolls, butter rolls and croissants, whole-grain rye bread, graham bread or rye bread with grains, graham, muesli, corn flakes with or without additives, whole-grain cereals, spelt flakes, oat flakes, barley flakes, fine-grained groats (e.g., semolina, cracked barley), wheat pasta, white rice, coarse-grained groats (e.g., buckwheat groats, pearl barley, brown rice, and whole-wheat pasta)) accounted for 97.65% (*n* = 83) in the study group and 100% (*n* = 96) in the control group. The consumption of dark green leafy vegetables (spinach, kale, Swiss chard, beet greens, arugula, romaine lettuce, parsley, green cabbage, chicory) was 41.18% (*n* = 35) in the study group and 68.75% (*n* = 66) in the control group. Consumption of root and tuber vegetables (carrots, beets, celery root, potatoes, sweet potatoes, parsley root, turnips, radishes, parsnips) was 32.94% (*n* = 28) in the study group and 21.88% (*n* = 21) in the control group. Consumption of other vegetables (tomatoes, cucumbers, bell peppers, zucchini, onions, eggplant, squash, pumpkin, fresh corn, cauliflower, broccoli, white and red cabbage, Brussels sprouts, leeks, garlic, green beans, green peas, asparagus, iceberg lettuce) was 45.88% (*n* = 39) in the study group and 15.63% (*n* = 15) in the control group. The consumption of fruits and fruit juices (apples, bananas, pears, berries, apple juice, orange juice, and banana juice) accounted for 77.65% (*n* = 66) among children in the study group and 88.54% (*n* = 85) in the control group. Consumption of legumes and oilseeds (lentils, chickpeas, soybeans, beans, nuts, and seeds) was 44.71% (*n* = 38) in the study group and 4.17% (*n* = 4) in the control group. Consumption of meat, fish, and poultry (chicken, turkey, rabbit, pork, beef, veal, cod, tuna, salmon, mackerel) was 95.29% (*n* = 81) in the study group and 96.88% (*n* = 93) in the control group. Consumption of milk and dairy products (milk, yogurt, kefir, buttermilk, cottage cheese, hard cheeses) was 95.29% (*n* = 81) in the study group and 83.33% (*n* = 80) in the control group. Egg consumption in the study group was 45.88% (*n* = 39) and 52.08% (*n* = 50) in the control group. Statistically significant differences in food intake between the groups were observed for dark green leafy vegetables, other vegetables, fruits and fruit juices, legumes and oilseeds, and milk and dairy products. The distribution of intake for individual food groups, taking into account the dietary diversity score, is presented in Table 5.

Ordinal logistic regression analysis of the relationship between study group membership and the level of feeding difficulty (MCH-FS) and dietary diversity (DDS) showed statistically significant differences between groups, expressed as odds ratios (ORs). In the case of MCH-FS, the absence of feeding difficulty was significantly more common in the study group than in the control group (22.35% vs. 2.08%), which corresponded to significantly higher odds of its occurrence in the study group (OR = 14.29; 95% CI: 3.03–67.45; *p* < 0.001). The highest level of feeding difficulty was also observed more often in the study group (21.18%) than in the control group (7.29%), and this difference was statistically significant (OR = 3.45; 95% CI: 1.35–8.33; *p* = 0.006). Dietary diversity analysis showed that low DDS levels were more common in the study group than in the control group (32.94% vs. 15.63%), which was associated with significantly higher odds of its occurrence in the study group (OR = 2.63; 95% CI: 1.30–5.56; *p* = 0.006). A similar relationship was observed for high DDS levels, which were also more common in the study group (34.12% vs. 19.79%), with a statistically significant difference between the groups (OR = 2.08; 95% CI: 1.08–4.17; *p* = 0.029) (Table 6).

### 3.4. Food Selectivity and Dietary Diversity Index

Linear regression analysis showed no significant association between the MCH-FS score and DDS. The regression model was not statistically significant (R^2^ = 0.0004; F(1.179) = 0.078; *p* = 0.781). The regression coefficient for the MCH-FS was very low and statistically insignificant (B = 0.02; SE = 0.07; β = 0.021; t(179) = 0.28; *p* = 0.781).

### 3.5. Sensory Factors and the Level of the DDS Index and Feeding Difficulties Based on the MCH-FS

In terms of acceptable food consistency, no statistically significant differences were found between groups for the preference for a solid, compact consistency with lumps depending on the DDS level (*p* > 0.05). The highest percentage of children in the study group accepting this consistency was observed with a low DDS (61.11%), while the values were similar to a medium and high DDS (42.86% and 50%). A statistically significant relationship was found for a solid, compact consistency without lumps (*p* = 0.036). In the control group, children with a medium DDS were more likely to accept this consistency (75.71%) than those in the study group (24.29%). At a low and high DDS, the distribution of responses between the groups was more even.

For the consistency of puree without lumps and liquid consistency, no statistically significant differences were found between the groups (*p* > 0.05). All children who accepted liquid consistency were included in the study group (100%), regardless of their DDS level. The analysis of acceptance of meals with a specific appearance did not reveal a significant relationship for affirmative responses (“yes”) (*p* = 0.094), with a tendency for such an appearance to be more frequently accepted in the study group with a high DDS (89.29%). However, statistical significance was observed for negative responses (“no”) (*p* = 0.014). In the control group, children with a medium DDS were more likely to disapprove of the specific appearance of meals (84.13%) compared to the study group (15.87%).

Regarding the acceptance of meals at a specific temperature, statistically significant differences were demonstrated for both the responses “yes—specified, measured” (*p* = 0.009) and “no—unspecified, unmeasured” (*p* = 0.001). Children in the study group more often declared acceptance of meals at a precisely specified temperature, especially with a low and medium DDS (100% of responses in the study group vs. 0% in the control group). In turn, the lack of temperature preference was more frequently observed in the control group, especially with a medium DDS (91.18% vs. 8.82% in the study group). Table 7 presents the relationship between children’s sensory profile and the level of the dietary diversity score (DDS: low, medium, high), taking into account the study and control groups.

In terms of accepted food consistency, no statistically significant differences were found for the preference for a solid, compact consistency with lumps (*p* = 0.206). The highest percentage of children accepting this consistency was observed in both the study and control groups at moderate and highest risk of feeding difficulties; however, the distribution of responses between the groups remained similar.

A significant correlation was found for a solid, compact consistency without lumps (*p* < 0.001). In the control group, particularly at moderate (70.59%) and moderate risk of feeding difficulties (81.43%), this consistency was more frequently accepted than in the study group (29.41% and 18.57%, respectively). However, at the highest risk of feeding difficulties, a higher percentage of children in the study group accepted this consistency (78.57%) compared to the control group (21.43%). No statistically significant differences were found for the consistency of puree without lumps and liquid consistency (*p* > 0.05). It is worth noting that only children in the study group (100%) accepted liquid consistency, regardless of the level of risk of feeding difficulties, while no such indications were noted in the control group.

Analysis of acceptance of meals with specific appearances revealed significant statistical differences for both affirmative (“yes”; *p* = 0.003) and negative (“no”; *p* = 0.001) responses. Children in the study group more often declared acceptance of meals with specific appearances, particularly in the absence of risk of feeding difficulties (100%) and moderate risk (66.67%). In contrast, the control group more often reported a lack of acceptance of specific meal appearances, especially in the moderate risk of feeding difficulties (88.71%).

Regarding the acceptance of meals at a specific temperature, no significant differences were found for the responses “yes—specific, measured” (*p* = 0.300). However, statistical significance was demonstrated for the responses “no—not specific, not measured” (*p* < 0.001). In the control group, children more often showed no preference for meal temperature, particularly in those with a medium (79.17%) and moderate risk of feeding difficulties (90.14%). In the study group, however, a preference for a specific temperature was more frequently observed, particularly in those with a moderate risk (96%). Table 8 below presents the relationship between sensory profile and the severity of feeding difficulties assessed using the MCH-FS (no risk, medium risk, moderate risk, highest risk), taking into account the study and control groups.

## 4. Discussion

Neurodevelopmental disorders, including autism spectrum disorder (ASD), are characterized by substantial clinical and behavioral heterogeneity. Feeding difficulties, food selectivity and atypical sensory responses are frequently reported in children with ASD, but their presentation and severity vary considerably between individuals [2,3]. Dietary behavior may therefore reflect the interaction of sensory characteristics, individual food preferences, developmental factors and the family and mealtime environment rather than a single underlying mechanism. In this context, the present study examined feeding difficulties, dietary diversity and selected sensory characteristics in children with ASD compared with children without an ASD diagnosis.

The findings should be interpreted primarily in terms of associations rather than causal relationships. Given the cross-sectional design, the present study cannot determine whether specific sensory characteristics contribute to feeding difficulties or reduced dietary diversity, whether feeding experiences influence sensory-related food preferences, or whether both are influenced by other developmental, clinical or environmental factors. Accordingly, the observed relationships provide a basis for identifying potential patterns of co-occurrence that warrant further investigation rather than evidence of causal mechanisms.

Moderate to moderate feeding difficulties were characterized by the highest percentage of six-year-olds and seven-year-olds in the study group, while moderate difficulties were observed in the control group in the same age range, with no significant differences between groups. Moderate difficulty with eating meals was found among the highest percentage of 10-year-olds in the control group of children without an ASD diagnosis. Descriptive assessment of individual factors on the MCH-FS revealed differences in the course of the meal, assessment of the child’s appetite (feeling of hunger), timing of meal refusal, behavior during meals, choking, spitting up, choking on certain foods, and forcing to eat and drink. Children from the study group were characterized by a more difficult mealtime, low appetite reported by parents, a quick moment of refusal to eat, picky, fussy behavior during meals, increased symptoms of choking, gagging and spitting out certain foods and frequent forcing of children to eat and drink by parents compared to the control group—children with ASD are about three times more likely to exhibit difficult eating behaviors, refuse products and have problems with their acceptance [8]. The above results partially confirm the study conducted by van Dijk et al. [16], in which the level of feeding difficulties increased with age in the control group (higher rate in the group of four- and six-year-olds than in the group of two-year-olds), and additionally in the analysis conducted by Bąblik et al. [4] and Dijk et al. [16] in terms of the greatest differences in eating difficulties (items 1, 3, 4—Bąblik et al. [4] and 1, 4, 6—Dijk et al. [16]) noted between the groups also concerned the assessment of the course of the meal, the moment of meal refusal, feeling of hunger and behavior during the meal, according to our analysis. Different results were obtained in terms of meal duration, because in our analysis and the study by Sforza et al. [17] conducted on the population of Italian children, the median duration of meals was 21–30 min, while in the study by van Dijk et al. [16] the duration was 11–20 min, it was shorter in the group of children with ASD. Based on our own results and the studies by Sforza et al. [17] and Bąblik et al. [4], this probably suggests food selectivity and aversion to meals, as well as the presence of distractors in the environment of the child with ASD, which is also confirmed by the study by Martins et al. [18], indicating picky eating behaviors in the group of children with ASD compared to siblings from the control group. Comparing this aspect based on our own interpretations, in the control group the duration of the meal process was 1–10 min, but in the analysis by van Dijk et al. [16] it was 11–20 min, which may indicate a lower degree of difficulty in accepting the color, smell, and temperature of products and meals in the observed control population [4,16,17,18].

The association between nutritional requirements (as assessed by the MCH-FS) and the study groups did not remain statistically significant after adjusting for age, sex, and BMI (β = −1.91; *p* = 0.056). Although the regression coefficient was notable and the *p*-value approached the threshold of significance, this result should not be interpreted as evidence of a lack of association between ASD and nutritional difficulties.

An analysis restricted to children aged ≤ 6 years also failed to show a statistically significant association between ASD status and MCH-FS scores (β = −3.34; *p* = 0.116). It should be noted that the small sample size (*n* = 59) limited the statistical power of this analysis. Consequently, while no significant effect was observed, the findings do not rule out an association between ASD and nutritional issues as measured by the MCH-FS. An interesting finding of the study was the absence of a significant difference between the groups regarding the total MCH-FS score, despite the presence of differences in specific questionnaire items. These results are not necessarily contradictory. The total MCH-FS score is an aggregate measure encompassing various aspects of feeding difficulties, whereas individual items relate to more specific behaviors and problems. Consequently, differences observed in isolated areas may not translate into a difference in the overall severity of feeding difficulties. This may also indicate that the nature of feeding difficulties in ASD is more specific and varied than an analysis based solely on the total questionnaire score would suggest. The lack of a significant association between the MCH-FS score and the DDS in the regression analysis also requires cautious interpretation. Although there are theoretical grounds to expect that greater feeding difficulties might be linked to a more limited dietary variety, dietary diversity is the result of the interplay of multiple factors. In addition to feeding difficulties, factors such as the child’s sensory preferences, the family environment, mealtime organization, the availability of and exposure to various foods, and prior dietary experiences and interventions may play a role. It is therefore possible that the relationship between feeding difficulties and dietary diversity is more complex and not simply linear. It should also be emphasized that a lack of statistical significance in the regression analysis does not equate to confirmation of the absence of a relationship between the variables analyzed. In the present study, the model did not show a significant association between the MCH-FS score and DDS; however, this result should be interpreted in the context of the study’s cross-sectional design, the heterogeneity of the study population, and the limited range of variables included in the model. Future studies should account for potential confounding and modifying factors and consider a more detailed analysis of the specific dimensions of feeding difficulties.

The present findings do not support a uniform reduction in dietary diversity among children with ASD. The primary analysis of the continuous DDS did not demonstrate a statistically significant difference between the ASD and control groups (*p* = 0.172). However, analysis of DDS categories revealed a more heterogeneous distribution within the ASD group, with children more frequently represented in both the low- and high-DDS categories compared with controls. In contrast, the control group was predominantly represented in the medium-DDS category. These findings suggest that dietary diversity among children with ASD may be characterized by greater within-group variability rather than by a consistent reduction in dietary diversity. Although ASD group membership was significantly associated with higher DDS after adjustment for age, sex, and BMI, the model explained only a modest proportion of the variance in the DDS (R^2^ = 0.078; adjusted R^2^ = 0.057). Therefore, other unmeasured factors likely contribute substantially to individual differences in dietary diversity. The Dietary Diversity Score allows for a qualitative assessment of dietary diversity within 9 food groups. The overall dietary assessment in our study indicates an average level of dietary quality in both the study group and the study group ($\overset{-}{X}=$ 7 years) and control ($\overset{-}{X}$ = 8 years) *n* the general population from 2 to 10 years of age (except for a significant difference in the median DDS at the age of 9 between the ASD and non-ASD groups; *p* = 0.054). However, no significant differences were observed between the groups in the quality of diet based on the DDS (Me = 6—ASD vs. Me = 5—non-ASD; *p* = 0.172), which is confirmed by the analyses conducted by Sağlam et al. (Me = 6.66—ASD Me = 7.08—non-ASD; *p* = 0.224) and the lack of significant differences between the groups of Turkish children with ASD ($\overset{-}{X}$ = 9 years) and their peers without a diagnosis ($\overset{-}{X}$ = 7 years) in the age range from 2 to 12 years of age [8]. Categorization of the dietary diversity score allowed us to obtain in our analyses the highest percentage of comparably low (32.94%; *n* = 28 study vs. 15.63%; *n* = 15 control) and medium (32.94%; *n* = 28 vs. 64.58%; *n* = 62) and high (34.12%; *n* = 29 vs. 19.79%; *n* = 19) feeding quality index, which indicates the diversity of feeding problems occurring in the group of children with ASD compared to their peers. The study conducted by Sağlami et al. indicates that low (14%; *n* = 7 study participants vs. 12%; *n* = 6 control participants), medium (26%; *n* = 13 study participants vs. 22%; *n* = 11 control participants), high dietary diversity score (60%; *n* = 30 study participants vs. 66%; *n* = 33 control participants) was not significantly different between the groups of children with ASD and without ASD diagnosis (*p* = 0.824), also suggesting, as in our own analysis, a different nutritional and sensory profile in the group of children with autism spectrum disorder, despite the different origin of the population of the studied children. Moreover, it was associated with difficulties such as irregular, excessive (32%) and rapid (38%) consumption of nutritionally selective meals (44%), refusal (40%), fits of anger and crying during meals (42%), significant in the study group compared to the control group (*p* < 0.05) [8].

Dietary patterns assessed based on our own qualitative analyses suggest a significantly increased consumption of dark green leafy vegetables (82.86%; *p* < 0.001) and other vegetables such as tomatoes, zucchini, peppers, and cauliflower (74.36%; *p* < 0.001) in the group of children with ASD compared to the control group. The above results do not correlate with studies on diet quality conducted by Gray et al. and Harris et al., which indicate different, undifferentiated dietary patterns in qualitative analysis in terms of low consumption of green and tuberous vegetables, legumes, fruits, and cereal products [19,20]. This discrepancy in comparison to other studies may result from the research methods used and the diverse population in terms of environmental and ethnic background. The different results of our study may suggest the heterogeneity of the population of children with ASD without the possibility of a uniform qualitative assessment of their nutritional habits, which constitutes a dietary therapeutic challenge in the interdisciplinary care model.

The absence of a statistically significant association between the MCH-FS and DDS in the present model suggests that dietary diversity cannot be adequately explained by feeding difficulties alone. Other factors, including sensory preferences, individual food acceptance, family feeding practices and previous dietary experiences, may also contribute to variation in the DDS. One possible explanation is that dietary diversity may be influenced by parental feeding practices and other adaptive strategies; however, these factors were not directly assessed in the present study, such as rotating accepted foods or modifying the presentation of meals. Furthermore, significantly lower odds of experiencing both no difficulties (OR = 14.29; 95% CI: 3.03–67.45; *p* < 0.001) and the most severe difficulties (OR = 3.45; 95% CI: 1.35–8.33; *p* = 0.006) were demonstrated in the control group compared to the study group. These findings indicate an association between ASD group membership and the distribution of feeding difficulty categories in the present sample. However, the clinical relevance of these differences should be interpreted cautiously and requires confirmation in larger studies. However, analysis of the dietary diversity score, at a low (OR = 2.63; 95% CI: 1.30–5.56; *p* = 0.006) and high DDS (OR = 2.08; 95% CI: 1.08–4.17; *p* = 0.029), was significantly less frequently observed in the control group compared to the study group. The analyses indicate that the distribution of dietary diversity differs between groups and may potentially be related to clinical or developmental status.

A different picture emerged regarding dietary diversity. In the multivariate analysis, ASD group membership remained significantly associated with the total DDS even after adjusting for age, sex, and BMI (β = 1.07; 95% CI: 0.44–1.70; *p* < 0.001). This finding suggests that the observed difference in dietary diversity was not solely a consequence of differences in age, sex, or BMI between the study groups.

At the same time, the regression model explained only 7.8% of the total variance in the DDS (R^2^ = 0.078), indicating that the majority of the variation in dietary diversity remained unexplained by the variables included in the model. The DDS could have been influenced by factors such as sensory issues, individual food preferences, the severity of food selectivity, the family environment, parental feeding practices, and other clinical characteristics of children with ASD that were not included in the current model. It should be emphasized that the significant association between ASD and the DDS cannot be interpreted as a cause-and-effect relationship. Given the cross-sectional nature of the study, one can only speak of an association between group membership and the DDS after adjusting for age, sex, and BMI.

Our analyses also revealed a significant relationship between the sensory profile of children on the autism spectrum and the dietary diversity index. Children with ASD presented a diverse dietary quality index, significantly more often correlated with the acceptance of solid, compact, lump-free consistency. Among children on the autism spectrum, food selectivity based on the DDS correlated with the acceptance of meals at a specific, previously measured temperature, while in the control group it correlated with the acceptance of unmeasured meal temperature. As well as significantly more frequent acceptance of meals after a previously unspecified, specific appearance among children without an ASD diagnosis. The above results are consistent with findings reported by Martins et al., Sağlam et al. dietary diversity does not directly reflect feeding difficulties but is likely the effect of long-term eating habits., Yeung et al., Harris et al., indicating a higher level of rigidity of eating behaviors, difficulties in the context of refusal, regularity of meal consumption, hypersensitivity, undersensitivity and sensory seeking among children with a spectrum of neurodevelopmental disorders [8,18,20,21]. When interpreting the results regarding sensory preferences, it should be noted that multiple statistical comparisons were performed without applying a correction for multiple testing. This increases the risk of a Type I error, and thus the probability that some of the statistically significant results observed may be due to chance. Therefore, individual statistically significant differences should be interpreted with caution and regarded as exploratory findings requiring confirmation in future studies. These results should not be interpreted as definitive evidence of differences in sensory preferences between children with ASD and the control group.

The relationship between sensory characteristics and dietary diversity warrants cautious interpretation. In the present study, selected sensory characteristics were associated with differences in dietary diversity, particularly in relation to food consistency, temperature and appearance. These findings are consistent with previous research indicating that sensory properties of food may be relevant to food acceptance and selective eating in children with ASD. However, the present study cannot establish whether sensory characteristics contribute to differences in dietary diversity or whether the observed associations reflect other factors influencing eating behavior. The literature suggests that food selectivity in ASD is a multidimensional phenomenon. Sensory sensitivity may coexist with behavioral rigidity, restricted food preferences, difficulties with food acceptance and individual patterns of exposure to foods. These factors may interact with family feeding practices, mealtime routines and previous therapeutic experiences. Thus, sensory characteristics should not be considered in isolation when interpreting dietary diversity. The heterogeneity observed in the present study is particularly relevant in this context. Children with ASD were not uniformly characterized by low dietary diversity; rather, low, medium and high DDS values were observed within the ASD group. This variability suggests that the relationship between sensory characteristics and dietary diversity may differ between individual children. The findings therefore support an individualized rather than uniform interpretation of feeding behavior in ASD. Future studies should investigate these potential profiles using longitudinal designs and models incorporating sensory, behavioral, developmental and family-related factors. The study by Molina-López et al. confirms that children with ASD have greater deficiencies in food intake (50% ASD, *p* = 0.014), a high level of food selectivity according to the FFQ—Food Frequency Questionnaire (60.6% ASD, *p* = 0.037) and significantly more eating problems in the form of food aversion and limited dietary diversity compared to neurotypical children (*p* = 0.001), while the study by João et al. confirms a specific, atypical sensory profile of children with ASD in the form of orofacial hypersensitivity (approximately 50% of cases) [22,23].

The results indicate that dietary diversity among children with ASD is not a uniform characteristic of this population. Although children with low DDS scores were observed within the ASD group, a significant portion of the study group achieved scores indicating high dietary diversity. This suggests considerable heterogeneity in dietary patterns within the ASD population, indicating that ASD-related feeding difficulties do not necessarily result in restricted dietary diversity for every child. The observed variability may be linked to numerous factors. Potential contributing factors include family dietary patterns and mealtime organization, prior nutritional or therapeutic interventions, the individual severity of feeding difficulties, and age-related developmental changes. However, as this study did not directly analyze the impact of these factors on the DDS, the mechanisms discussed should be viewed as potential explanations for the observed heterogeneity.

The observed relationship between age and the DDS in the ASD group also warrants particular attention. A positive correlation suggests that, for some children, dietary diversity may increase with age. However, this pattern does not apply to all children with ASD, and the cross-sectional nature of the study precludes drawing conclusions about longitudinal changes within the same individuals. From a clinical perspective, these findings highlight the need for an individualized approach to assessing the nutrition of children with ASD. An ASD diagnosis alone should not be considered sufficient grounds to assume the presence of limited dietary diversity. It may be more important to determine the child’s individual nutritional profile, encompassing, among other things, range of accepted foods, sensory preferences, mealtime difficulties, and the actual diversity of the diet consumed.

A crucial aspect of interpreting the results is the potential presence of confounding factors. The study groups may have differed in terms of demographic and anthropometric characteristics, and factors such as age, sex, and BMI can be associated with children’s eating behaviors. Consequently, relying solely on unadjusted between-group comparisons would not allow for an assessment of whether the observed differences were independently associated with ASD group membership.

Incorporating age, sex, and BMI into regression models made it possible to evaluate the associations between group membership and MCH-FS and DDS scores while accounting for these potential confounders. The results indicated that the association between group membership and DDS scores persisted after adjustment, whereas for MCH-FS scores, it did not reach statistical significance. These findings suggest that the influence of potential confounding factors may vary depending on the specific aspect of eating behavior being analyzed.

Overall, the findings suggest that feeding difficulties, sensory characteristics and dietary diversity should be considered as interconnected but not necessarily linearly related aspects of eating behavior in children with ASD. The observed heterogeneity indicates that neither feeding difficulties nor dietary diversity can be adequately characterized by a single pattern applicable to all children with ASD. From a clinical perspective, these findings support the value of individualized nutritional assessment that considers food selectivity, sensory preferences, mealtime behavior and actual dietary diversity.

## 5. Conclusions

1. The overall MCH-FS score did not differ statistically significantly between children with ASD and the control group, indicating no evidence of a significant between-group difference in the overall severity of feeding difficulties. However, statistically significant differences were observed for selected feeding behaviors, including very difficult mealtimes, sporadic hunger or lack of appetite, food refusal at the beginning of meals, picky and fussy eating behavior, frequent parental prompting to drink and eat, and selected adverse feeding responses. These findings suggest differences in specific feeding behaviors rather than a generalized difference in overall feeding difficulties.
2. Among children with ASD, selected sensory characteristics of food, particularly acceptance of solid, compact consistency without lumps, meal temperature, and food appearance, were significantly associated with the DDS. These findings suggest that sensory characteristics may be relevant to dietary diversity in some children with ASD. However, given the cross-sectional design and the exploratory nature of these analyses, the observed associations should not be interpreted as evidence of causal mechanisms. The absence of a significant association between the overall MCH-FS score and the DDS further suggests that dietary diversity is likely influenced by multiple factors beyond the overall severity of feeding difficulties.
3. The primary analysis of the continuous DDS did not demonstrate a statistically significant difference in overall dietary diversity between children with ASD and controls. However, the categorical analysis revealed greater heterogeneity within the ASD group, with children more frequently represented in both the low- and high-DDS categories, whereas the control group was predominantly represented in the medium-DDS category. Selected differences in the consumption of individual food groups were also observed, including legumes and oilseeds and selected vegetables, as well as lower consumption of milk and dairy products and fruits and fruit juices among children with a lower DDS. These findings should be interpreted as exploratory, particularly given the multiple comparisons performed, and require confirmation in future studies.

The study results indicate a heterogeneous pattern of dietary diversity among children with ASD, without confirming an overall reduced level. The unadjusted comparison of a continuous DDS did not show a statistically significant difference between groups; however, after adjustment for age, sex, and BMI, ASD group membership was significantly associated with a higher DDS. Given the modest explanatory value of the adjusted model, these findings should be interpreted cautiously and support the need for individualized assessment of dietary diversity and nutritional needs in children with ASD.

In summary, feeding problems assessed using the MCH-FS indicate a significant relationship between the degree of feeding behavior and modeling of feeding and sensory behaviors in terms of factors such as specific temperature, smell, and appearance (color, texture, consistency, presentation) of food and the entire meal among children with ASD.

The study indicates differences in selected aspects of eating behaviors between children with ASD and the control group. After adjusting for age, sex, and BMI, group membership (ASD vs. control) remained significantly associated with the DDS, whereas the association with the MCH-FS score did not reach statistical significance. The findings suggest that dietary diversity may differ between children with ASD and the control group, independent of the basic demographic and anthropometric factors included in the model. However, the cross-sectional nature of the study and the use of a convenience sample limit the ability to draw causal inferences or generalize the results. Future research should involve larger, more representative samples and consider a broader range of potential factors associated with the eating behaviors of children with ASD.

## 6. Practical Implications

**Figure 1:**
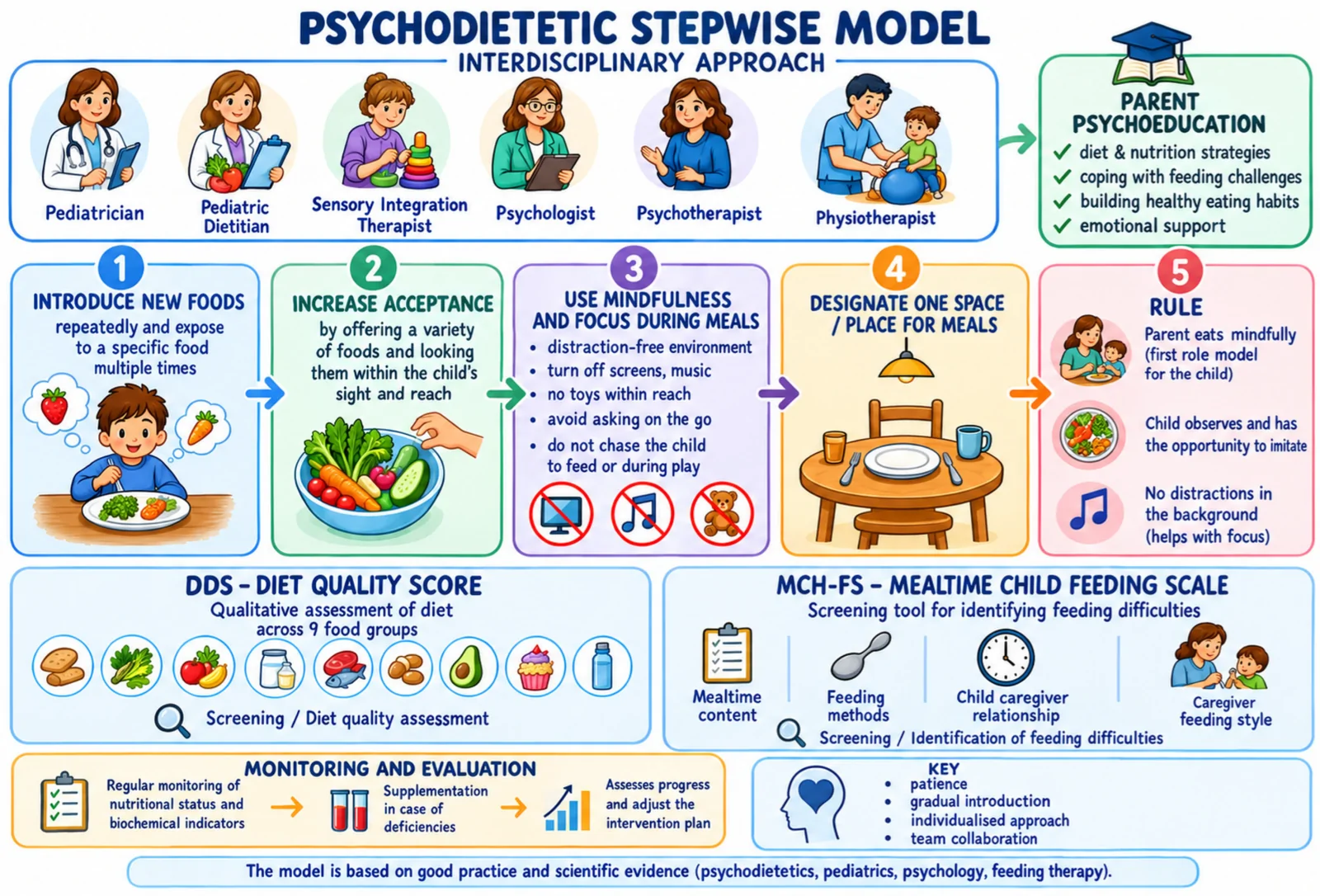
Our own psychodietetic model for managing food selectivity in children with autism spectrum disorder. Own work. Generated with OpenAI’s ChatGPT (GPT-5.2).

In clinical practice, the findings support an individualized and interdisciplinary approach to feeding and nutritional assessment in children with ASD. Such an approach may involve collaboration between a pediatrician, pediatric dietitian, sensory integration specialist, psychologist, psychotherapist, and physiotherapist, depending on the child’s individual needs. When introducing new foods, nutritional goals may be complemented by sensory-based strategies and gradual food exposure, including the food chain method. Given the heterogeneity of dietary diversity and feeding characteristics observed in the present study, interventions should be tailored to the individual child’s dietary pattern, feeding difficulties and sensory characteristics rather than based solely on the diagnosis of ASD. The proposed psychodietetic treatment model, introduced in Figure 1 (Figure 1), includes detailed information on the implementation of psychoeducation into the mandatory model, as adherence to education regarding the expansion of dietary principles is essential. Routine activities related to nutrition and biochemical criteria, as well as supplementation recommendations for nutritional deficiencies, are essential, as well as risk factors for nutritional deficiencies among children with autism spectrum disorder.

## 7. Strengths and Limitations of the Study

This study is the first to assess the relationship between food selectivity, dietary quality index, and sensory patterns in children with autism spectrum disorder (ASD) in the Polish population. A significant advantage of the project was the inclusion of a control group and the multidimensional analysis of dietary behaviors. However, the results should be interpreted with consideration of the study’s limitations, such as the cross-sectional nature of the project, reliance on parental self-report, and limited sample size. The limited representativeness of the sample means that the obtained results may not accurately reflect the entire population of children with ASD, but rather the specific group of individuals included in the study.

A convenience sampling method was used, which entails a risk of selection bias. Participants were recruited from clinical settings; therefore, their characteristics may differ from those of children with ASD or children in the general population. This limits the generalizability of the findings to the entire population of children aged 2–10 years.

The study was cross-sectional in design; consequently, the observed associations cannot be interpreted as cause-and-effect relationships. This also applies to the association between ASD group membership and the DDS that persisted after adjustment.

When interpreting the MCH-FS results, limitations regarding the age range for which the tool version was validated must be considered. To assess the stability of the findings, an additional sensitivity analysis was conducted, restricted to children aged ≤ 6 years. The direction of the effect was consistent with the analysis of the entire population, although the association did not reach statistical significance. This result should be interpreted in light of the considerably smaller sample size used in the sensitivity analysis.

Another limitation of the study was the use of a single 24 h dietary interview to assess food intake and dietary diversity. Although this method allows for a detailed reconstruction of food intake on a specific day, a single measurement may not reflect a child’s usual dietary patterns or typical dietary diversity, due to the natural variability in intake from day to day. Consequently, the DDS index obtained may be partially dependent on the specific day on which the interview was conducted and may not fully reflect long-term dietary patterns. In future studies, it would be appropriate to use repeated 24 h dietary interviews or multi-day food diaries, which would allow for a more reliable assessment of habitual intake and dietary diversity. The relatively large number of statistical tests increases the possibility of Type I error. Therefore, individual statistically significant findings from these analyses should be considered exploratory and require confirmation in future studies with prespecified hypotheses and appropriate control of the family-wise error rate or false discovery rate.

Despite adjusting for age, sex, and BMI in the multivariate analysis, the influence of other potential confounding factors cannot be ruled out. In particular, the models did not account for all possible factors related to eating behaviors, such as the severity of ASD symptoms, sensory difficulties, parental feeding practices, or specific characteristics of food selectivity. In the future, longitudinal studies with larger groups of participants, including detailed assessment of nutritional status and quantitative nutrient intake, seem warranted.

## Figures and Tables

**Table 1:** Characteristics of the study and control groups.

Variable	ASD (*n* = 85)	Control (*n* = 96)	the Value of *p*
Age [years] *	7	8	0.005
Girls *n* (%)	28 (33.33%)	56 (66.67%)	0.001
Boys *n* (%)	57 (58.76%)	40 (41.24%)	
Body weight [kg] *	27.06	39.04	<0.001
Body height [cm] *	125.12	131.30	0.027
BMI [kg/m^2^] *	16.05	21.69	<0.001
Underweight *n* (%)	25 (83.33%)	5 (16.67%)	<0.001
Normal body weight *n* (%)	46 (64.79%)	25 (35.21%)	
Overweight *n* (%)	6 (25%)	18 (75%)	
Obesity *n* (%)	8 (14.29%)	48 (85.71%)	

* average value of the numerical data.

**Table 2:** Comparison of the main components of the MCH-FS between the study group and the control group.

Question	Factor	Study Group *Mean* (*Standard Deviation*)	Control Group *Mean* (*Standard Deviation*)	Study Group *Mediana*	Control Group *Mediana*
**1. How would you rate your child’s mealtime experience?**	course of the meal	3.15 (±1.84)	5.73 (±1.75)	2	7
**2. How concerned are you about your child’s mealtimes?**	parents’ concerns	4.73 (±2)	3.65 (±1.42)	6	4
**3. How would you rate your child’s appetite (sense of hunger)?**	a child’s appetite	3.66 (±2.15)	5.89 (±1.71)	3	7
**4. At what point during a meal does a child start refusing to eat?**	the moment of refusing meals	2.64 (±2.01)	5.47 (±2.27)	1	7
**5. How long do your child’s meals last (in minutes)?**	meal duration	3.40 (±1.68)	1.68 (±0.99)	3	1
**6. How would you rate your child’s behavior during meals?**	table manners	5.28 (±2.03)	2.29 (±1.61)	6	2
**7. Does your child choke, gag, spit out, or vomit when eating certain types of food?**	choking, gagging, spitting out certain foods	4.67 (±2.19)	1.93 (±1.74)	6	1
**8. Does the child hold food in their mouth without swallowing it?**	holding food in the mouth	3.48 (±2.52)	2.79 (±1.75)	2	2
**9. Do you have to follow your child around or distract them (with toys or TV) to get them to eat?**	distracting a child while eating	3.29 (±2.62)	2.04 (±1.68)	1	1
**10. Do you have to force your child to eat or drink?**	forcing someone to eat and drink	3.21 (±2.39)	5.91 (±1.88)	2	7
**11. How would you rate your child’s chewing (or sucking) skills?**	the ability to chew or suck	2.62 (±1.79)	2.36 (±0.88)	2	2
**12. How would you rate your child’s growth (weight, height)?**	a child’s growth	5.36 (±1.90)	6.44 (±1.09)	6	7
**13. How does feeding a child affect your relationship with them?**	parent–child relationships	3.59 (±1.96)	5.31 (±1.49)	2	6
**14. How does feeding a child affect family relationships?**	family relationships	3.22 (±1.82)	2.58 (±1.48)	2	2

**Table 3:** Age distribution of children and DDS scores in the study and control groups.

Age (Years)	Study Group (*n* = 85)	Median DDS (Q1–Q3)	Control Group (*n* = 96)	Median DDS (Q1–Q3)
**2**	2	5 (4–6)	4	6 (5–7)
**3**	0	-	6	5.5 (4–7)
**4**	6	5 (4–5)	5	8 (7–9)
**5**	12	4 (3.5–6)	4	6 (5–7.5)
**6**	18	5 (4–6)	2	5.5 (4–7)
**7**	15	6 (3–8)	9	5 (5–5)
**8**	7	5 (4–8)	8	5 (4.5–5)
**9**	14	6 (4–8)	22	5 (4–5)
**10**	11	7 (7–8)	36	5 (5–5)

Due to the small sizes of some age subgroups, only descriptive statistics are presented; no separate between-group comparisons were performed for individual age categories.

**Table 4:** Comparison of food product groups based on the DDS index, broken down by test and control groups.

Food Product Group	Study Group *Mean* *(Standard Deviation)*	Control Group *Mean* *(Standard Deviation)*	Study Group *Median*	Control Group *Median*	the Value of *p*
**1—grains**	0.98 (±0.15)	1 (±0.00)	1	1	0.132
**2—dark green leafy vegetables**	0.41 (±0.50)	0.69 (±0.47)	0	1	<0.001
**3—root and tuber vegetables**	0.33(±0.47)	0.22 (±0.42)	0	0	0.095
**4—other vegetables**	0.46 (±0.50)	0.16 (±0.36)	0	0	<0.001
**5—fruit and fruit juices**	0.78 (±0.42)	0.89 (±0.32)	1	1	0.051
**6—legumes and oilseeds**	0.45 (±0.50)	0.04 (±0.20)	0	0	<0.001
**7—meat/fish/poultry**	0.95 (±0.21)	0.97 (±0.17)	1	1	0.584
**8—milkand dairy products**	0.95 (±0.21)	0.83 (±0.37)	1	1	<0.010
**9—eggs**	0.46 (±0.50)	0.52 (±0.50)	0	1	0.408

**Table 5:** Consumption of individual food groups divided by dietary diversity score among the study and control groups.

Food Product Group	Low DDS		Medium DDS		High DDS		the Value of *p*
	Study Group *n* (%)	Control Group *n* (%)	Study Group *n* (%)	Control Group *n* (%)	Study Group *n* (%)	Control Group *n* (%)	Study Group vs. Control Group
**1—grains**	26	15	28	62	29	19	0.219
	(31.33%)	(15.63%)	(33.73%)	(64.58%)	(34.94%)	(19.79%)	
**2—dark green leafy vegetables**	0	17	6	0	29	49	<0.001
	(0.00%)	(25.76%)	(17.14%)	(0.00%)	(82.86%)	(74.24%)	
**3—root and tuber vegetables**	1	18	7	1	20	2	0.066
	(3.57%)	(85.71%)	(25.00%)	(4.76%)	(71.43%)	(9.52%)	
**4—other vegetables**	0	12	10	1	29	2	<0.001
	(0.00%)	(80.00%)	(25.64%)	(6.67%)	(74.36%)	(13.33%)	
**5—fruit and fruit juices**	22	19	24	11	20	55	0.039
	(33.33%)	(22.35%)	(36.36%)	(12.94%)	(30.30%)	(64.71%)	
**6—legumes and oilseeds**	1	2	18	0	19	2	<0.001
	(2.63%)	(50.00%)	(47.37%)	(0.00%)	(50.00%)	(50.00%)	
**7—meat/fish/poultry**	24	19	28	12	29	62	0.433
	(29.63%)	(20.43%)	(34.57%)	(12.90%)	(35.80%)	(66.67%)	
**8—milk and dairy products**	26	19	26	14	29	47	0.009
	(32.10%)	(23.75%)	(32.10%)	(17.50%)	(35.80%)	(58.75%)	
**9—eggs**	2	14	11	0	26	36	0.247
	(5.13%)	(28.00%)	(28.20%)	(0.00%)	(66.67%)	(72.00%)	

*p* values were calculated using Fisher’s exact test.

**Table 6:** Relationship between study group membership and DDS and MCH-FS levels.

Variable	Study Group *n* (%)	Control Group *n* (%)	OR (95% CI)	*p* ch^2^NW
**MCH-FS no difficulty** vs. other categories	19	2	14.29	<0.001
	(22.35%)	(2.08%)	(3.03–67.45)	
**MCH-FS highest difficulty** vs. other categories	18	7	3.45	0.006
	(21.18%)	(7.29%)	(1.35–8.33)	
**DDS low** vs. other categories	28	15	2.63	0.006
	(32.94%)	(15.63%)	(1.30–5.56)	
**DDS high** vs. other categories	29	19	2.08	0.029
	(34.12%)	(19.79%)	(1.08–4.17)	

**Table 7:** Sensory conditions related to food, taking into account the level of the dietary diversity score.

Acceptable Consistency	Low DDS		Medium DDS		High DDS		*p* ch^2^NW	
	Study Group *n* (%)	Control Group *n* (%)	Study Group *n* (%)	Control Group *n* (%)	Study Group *n* (%)	Control Group *n* (%)	Study	Control
**Solid, compact, with lumps**	11	7	6	8	6	6	0.579	
	(61.11%)	(38.89%)	(42.86%)	(57.14%)	(50%)	(50%)		
**Solid, compact, no lumps**	7	7	17	53	12	13	0.036	
	(50%)	(50%)	(24.29%)	(75.71%)	(48%)	(52%)		
**Puree, no lumps**	3	1	0	1	3	0	0.105	
	(75%)	(25%)	(0%)	(100%)	(100%)	(0%)		
**Liquid**	7	0	5	0	8	0	1.000	
	(100%)	(0%)	(100%)	(0%)	(100%)	(0%)		
**Acceptance of meals of a specific appearance**								
**Yes**	20	8	18	9	25	3	0.094	
	(71.43%)	(28.57%)	(66.67%)	(33.33%)	(89.29%)	(10.71%)		
**No**	8	7	10	53	4	16	0.014	
	(53.33%)	(46.67%)	(15.87%)	(84.13%)	(20%)	(80%)		
**Acceptance of meals at a specific temperature**								
**Yes—defined, measured**	18	0	22	0	23	5	**0.009**	
	(100%)	(100%)	(100%)	(0%)	(82.14%)	(17.86%)		
**Undefined, immeasurable**	10	15	6	62	6	12	**0.001**	
	(40%)	(60%)	(8.82%)	(91.18%)	(33.33%)	(66.67%)		

**Table 8:** Sensory conditions related to eating, including feeding difficulties (MCH-FS).

Acceptable Consistency	No Risk		Medium Risk		Moderate Risk		Highest Risk		*p* ch^2^NW	
	Study Group *n* (%)	Control Group *n* (%)	Study Group *n* (%)	Control Group *n* (%)	Study Group *n* (%)	Control Group *n* (%)	Study Group *n* (%)	Control Group *n* (%)	Study	Control
**Solid, compact, with lumps**	6	1	5	8	9	9	3	3	0.206	
	(85.71%)	(14.29%)	(38.46%)	(61.54%)	(50%)	(50%)	(50%)	(50%)		
**Solid, compact, no lumps**	7	1	5	12	13	57	11	3	<0.001	
	(87.50%)	(12.50%)	(29.41%)	(70.59%)	(18.57%)	(81.43%)	(78.57%)	(21.43%)		
**Puree, no lumps**	3	0	1	0	2	1	0	1	0.159	
	(100%)	(0%)	(100%)	(0%)	(66.67%)	(33.33%)	(0%)	(100%)		
**Liquid**	3	0	6	0	7	0	4	0	1.000	
	(100%)	(0%)	(100%)	(0%)	(100%)	(0%)	(100%)	(0%)		
**Acceptance of meals of a specific appearance**										
**Yes**	14	0	12	7	24	12	13	1	0.003	
	(100%)	(0%)	(63.16%)	(36.84%)	(66.67%)	(33.33%)	(92.86%)	(7.14%)		
**No**	5	2	5	13	7	55	5	6	0.001	
	(71.43%)	(28.57%)	(27.78%)	(72.22%)	(11.29%)	(88.71%)	(45.45%)	(54.55%)		
**Acceptance of meals at a specific temperature**										
**Yes—defined, measured**	11	0	12	1	24	1	16	3	0.300	
	(100%)	(0%)	(92.31%)	(7.69%)	(96%)	(4%)	(84.21%)	(15.79%)		
**No—undefined, immeasurable**	8	2	5	19	7	64	2	4	<0.001	
	(80%)	(20%)	(20.83%)	(79.17%)	(9.86%)	(90.14%)	(33.33%)	(66.67%)		

## Data Availability

The data presented in this study are available on request from the corresponding author due to privacy.
